# Combined Immune Checkpoint Blockade and Stereotactic Ablative Radiotherapy Can Stimulate Response to Immunotherapy in Metastatic Melanoma: A Case Report

**DOI:** 10.7759/cureus.4038

**Published:** 2019-02-08

**Authors:** Angel Moran, Soheila Azghadi, Emanual M Maverakis, Scott Christensen, Brandon A Dyer

**Affiliations:** 1 Radiation Oncology, University of California Davis Comprehensive Cancer Center, Sacramento, USA; 2 Dermatology, University of California Davis Comprehensive Cancer Center, Sacramento, USA; 3 Hematology & Oncology, University of California Davis Comprehensive Cancer Center, Sacramento, USA

**Keywords:** abscopal effect, melanoma, radiation therapy, stereotactic ablative radiotherapy, immunotherapy, pd-1

## Abstract

Skin cancer is the most commonly diagnosed malignancy in the United States, and invasive cutaneous melanoma is responsible for the vast majority of skin cancer-related deaths. Treatment options for patients with regional nodal disease, in-transit metastases, or locally advanced or distant metastatic disease are challenging. Historically survival rates in this patient population are dismal. Improved systemic control is possible using targeted agents and checkpoint inhibitors have redefined treatment outcomes. Furthermore, multi-modal therapy incorporating radiation may improve survival outcomes by priming the immune system for antigen release and help in reversing T-cell exhaustion. Herein, we describe a patient with widespread metastatic melanoma with progressive systemic disease while receiving checkpoint inhibition therapy that was reversed after combined immunoradiotherapy. The patient is now more than 41 months from diagnosis with durable, stable systemic disease.

## Introduction

Skin cancer is the most commonly diagnosed malignancy in the United States (US). Invasive cutaneous melanoma accounts for only 1% of all skin cancer cases, however, it is responsible for the vast majority of skin cancer-related deaths. In 2018, an estimated 91,270 new cases of melanoma will be diagnosed and an estimated 9,320 deaths will occur [1]. Malignant melanoma is most commonly diagnosed in non-Hispanic Caucasians with an annual incidence rate of 26 per 100,000. Furthermore, the incidence of malignant melanoma skin malignancies has risen rapidly over the past 30 years [1].

Treatment for localized disease includes wide local excision, possibly with sentinel lymph node biopsy. For patients with regional disease involvement, or particularly unresectable and/or distant disease, there have been no significant changes in medical management through the first decade of the 21^st^ century. In patients with distant metastatic disease, one year survival rates are 62% for non-lung soft tissue disease (M1a), 53% for those with lung disease (M1b), and 33% for non-central nervous system (CNS) visceral disease (M1c) [2]. However, with advances in the understanding of molecular mechanisms and targeted cell cycle checkpoint inhibitors, immunotherapy strategies have radically transformed the care for patients with locoregional or metastatic melanoma. The development of targeted agents and immune-oncology (I/O) has resulted in major improvements in patient outcomes [3, 4]. Two major new classes of effective systemic therapeutic agents are now in widespread clinical use for melanoma, including checkpoint inhibitors against cytotoxic T lymphocytes antigen 4 (CTLA-4), and programmed death 1 (PD-1).

Herein, we describe a patient with metastatic melanoma with a remarkable clinical course following combined immune checkpoint blockade and stereotactic ablative radiotherapy. Written informed consent was obtained from the patient prior to drafting this text and no identifying information appears in this article.

## Case presentation

**Figure 1:**
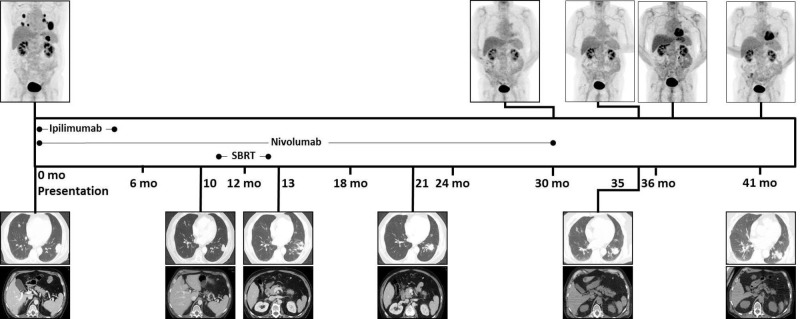
Treatment timeline. Representative staging and surveillance images from PET/CT MIP (above), and CT chest and abdomen (below) are shown, as well as timing of treatment received. PET/CT: Positron emission tomography/computed tomography; MIP: Maximum intensity projection.

**Figure 2:**
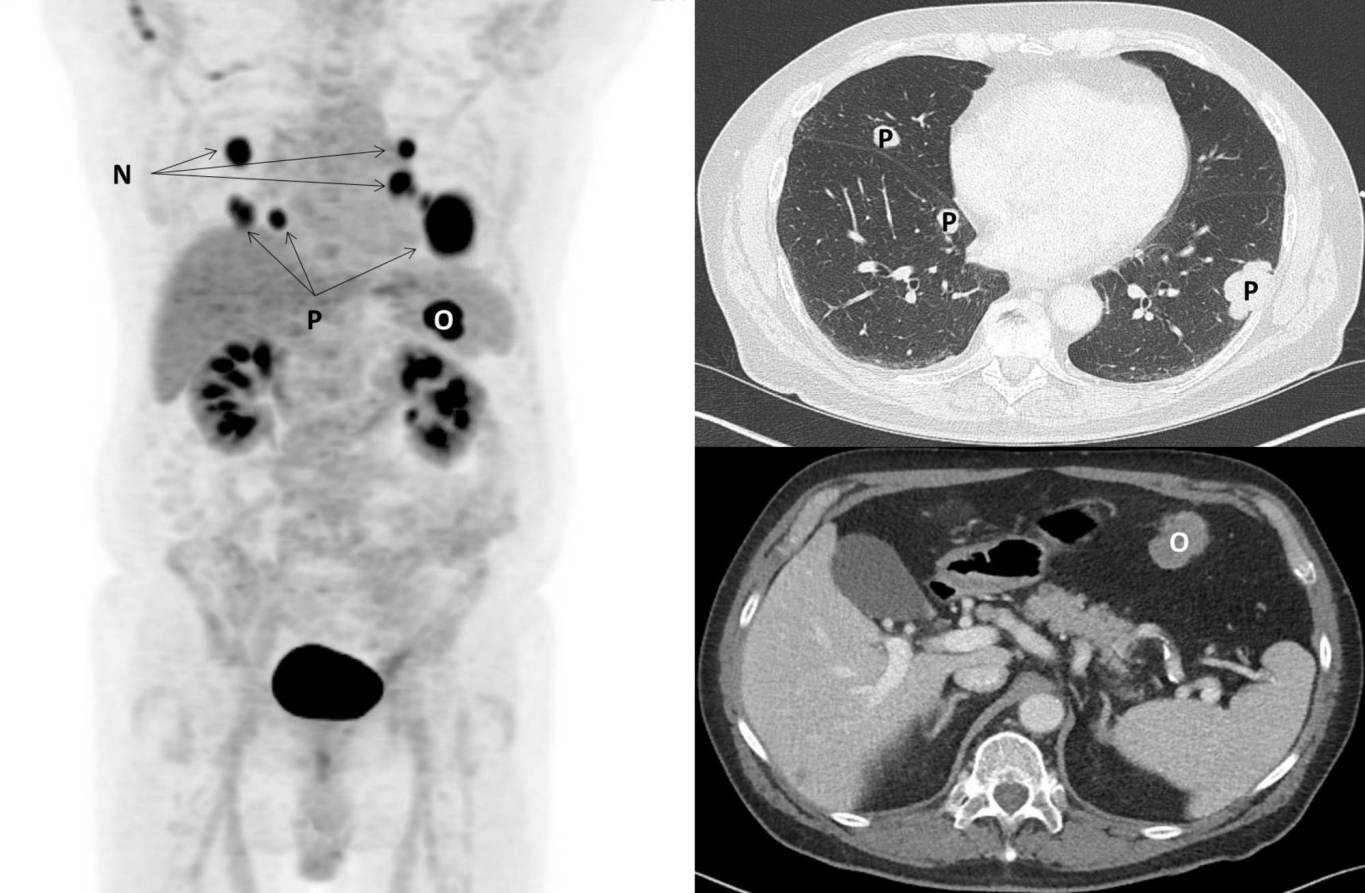
Staging imaging at diagnosis. PET/CT maximum intensity projection (left), and representative transverse CT chest (right, top) and CT abdomen (right, bottom) images are shown. Multiple FDG-avid bilateral hilar lymph nodes (N; left) and pulmonary parenchymal nodules (P; left, and right, top) are seen. An FDG-avid omental mass (O; left, and right, bottom) is seen. PET/CT: Positron emission tomography/computed tomography; FDG: Fluorodeoxyglucose.

A 71-year-old man with a history of multiple non-melanoma skin cancers and an ascending aortic aneurysm completed chest computed tomography (CT) for cardiopulmonary surveillance on 5/27/2015 and was found to have multiple bilateral lung masses. Subsequent CT abdomen and pelvis demonstrated a 4 cm omental mass concerning for malignancy. Systemic staging with fluorodeoxyglucose (FDG) positron emission tomography (PET)/CT demonstrated intense FDG-avidity of the lung masses, omental mass, and bilateral hilar nodes (Figures 1, 2). A CT-guided biopsy of a left lung lower mass demonstrated poorly differentiated metastatic malignant melanoma. Immunohistochemical staining (IHC) was positive for: S100, and Melan A, and negative for: TTF1, P63, and CK7/20. Given severe claustrophobia, a CT head was performed and demonstrated no brain metastases.

Taken together, based on AJCC8, the patient had cTxNxM1c (stage IV) melanoma [2]. Genetic testing revealed no mutations in the BRAF gene. Intravenous (IV) systemic therapy was initiated with dual checkpoint blockade using ipilimumab (3 mg/kg) and nivolumab (3 mg/kg) given every three weeks.

After one cycle of dual checkpoint blockade, the patient had multiple grade 1-3 side effects which were felt to be ipilimumab-related, and ipilimumab was subsequently discontinued. After three cycles of nivolumab, four months from diagnosis, surveillance CT imaging showed interval partial response (PR) of the pulmonary parenchymal metastases and hilar lymph nodes. After nine additional cycles of nivolumab, seven months from diagnosis, surveillance CT imaging demonstrated continued PR of the right lung and omental metastases. Additional sites of metastatic disease were stable (SD) and no new metastases were identified.

After 20 cycles of nivolumab, 10 months from diagnosis, surveillance CT imaging demonstrated interval enlargement of two left lower lobe pulmonary nodules, and several left hilar lymph nodes (Figure 1). The omental metastasis had radiographic near complete response (CR) and no new sites of metastatic disease were identified.

After 35 cycles of nivolumab, 12 months from diagnosis, the patient completed stereotactic body radiation therapy (SBRT) to the two enlarging left lower pulmonary nodules to 50 Gray (Gy) in five fractions. One month later, 13 months from diagnosis, surveillance CT imaging demonstrated interval improvement of metastatic disease in the chest (PR) and no evidence of metastatic disease in the abdomen/pelvis (Figure 1).

**Figure 3:**
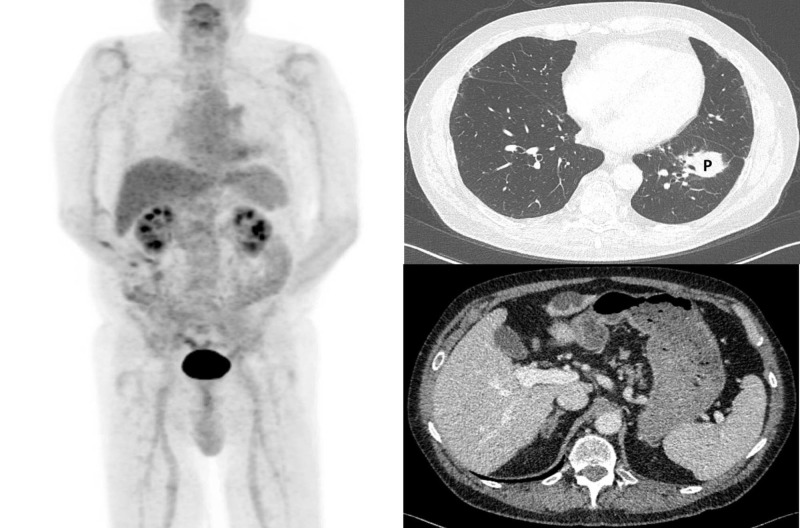
Surveillance imaging after completion of nivolumab and left lung SBRT at 30 months from diagnosis. PET/CT maximum intensity projection (left), and representative transverse CT chest (right, top) and CT abdomen (right, bottom) images are shown. There is no evidence of metabolically active disease. The left lung parenchymal lesions treated with SBRT are stable in size and morphology (P; right, top). The initially seen right-sided pulmonary parenchymal nodules have demonstrated stable partial response. The omental mass has radiographically resolved (right, bottom). PET/CT: Positron emission tomography/computed tomography; SBRT: Stereotactic body radiation therapy.

After 43 cycles of nivolumab, 21 months from diagnosis, surveillance CT imaging demonstrated PR in the chest, and no evidence of disease in the abdomen/pelvis. After 60 cycles of nivolumab, 30 months from diagnosis, surveillance PET/CT imaging showed systemic metabolic CR with continued stable PR in the chest and no evidence of metastatic disease in the abdomen/pelvis (Figures 1, 3). Plans for additional systemic therapy with nivolumab were discontinued.

**Figure 4:**
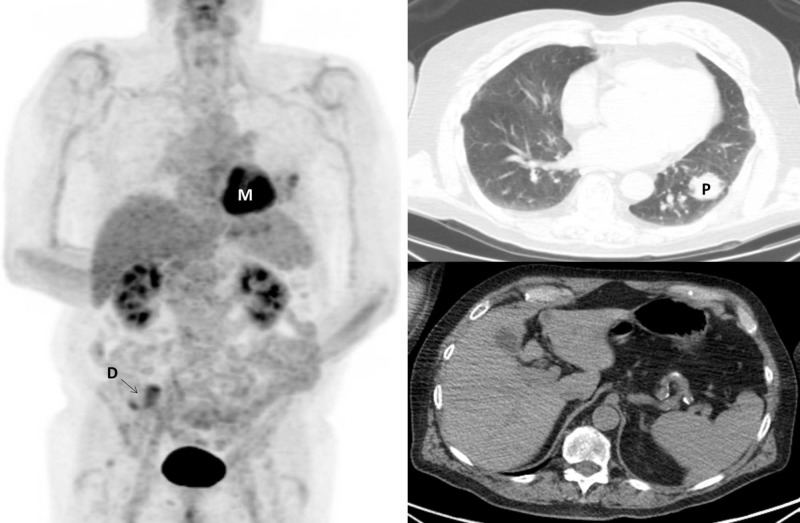
Surveillance imaging at 41 months from diagnosis. PET/CT maximum intensity projection (left) and representative transverse CT chest (right, top) and CT abdomen (right, bottom) images are shown. The myocardium is FDG-avid (M; left) and the patient had mildly avid diverticulosis (D; left). There is no evidence of metabolically active disease. The left lung parenchymal lesions treated with SBRT (P; right, top) are stable in size and morphology (right, top). The initially seen right-sided pulmonary parenchymal nodules have demonstrated stable partial response. The omental mass has radiographically resolved (right, bottom). PET/CT: Positron emission tomography/computed tomography; FDG: Fluorodeoxyglucose; SBRT: Stereotactic body radiation therapy.

PET/CT imaging completed five months later after discontinuation of nivolumab, 35 months from diagnosis, demonstrated interval increase in left hilar FDG activity; however, repeat PET/CT imaging three months later demonstrated decreased FDG activity in this area and no new metastases. The patient is now 41 months from diagnosis and recent surveillance imaging is stable, without evidence of FDG-avid disease or new metastases (Figures 1, 4).

## Discussion

Herein, we discussed the case of a patient with widespread metastatic melanoma with initial mixed response to immunotherapy, then with durable, complete metabolic systemic response following SBRT to two lung metastases. The patient is now more than 41 months from their initial diagnosis with ongoing durable clinical response. Based on AJCC8 staging and survival data, the estimated one-year overall survival rates in patients with metastatic melanoma are 62% for non-lung soft tissue disease (M1a), 53% for lung disease (M1b), and 33% for non-CNS visceral disease (M1c) [2].

Melanoma is highly immune-mediated and malignant lesions are associated with immune infiltrates, such as tumor infiltrating lymphocytes (TILs). TILs are implicated in improved clinical response, therapeutic efficacy, and destruction of solid tumors – specifically in metastatic melanoma [5, 6]. The finding that TIL positive disease is associated with improved patient outcomes is suggestive that intratumoral TILs (adaptive immune resistance) may represent a useful treatment stratification variable and provides further rationale for immunotherapeutic approaches. These findings highlight the need to explore and develop novel biomarkers and targets for combined therapeutic approaches.

With respect to T-cell clonality, increased T-cell receptor (TCR) clonality at baseline and expansion of individual clones with treatment has been associated with improved outcomes in patients with metastatic melanoma [7]. When TCR diversity was evaluated in response to radiotherapy in animal models the overall intratumoral diversity increased after radiotherapy and, when combined with CTLA-4 blockade, abscopal effects were demonstrated [8].

Radiotherapy is optimal for combined modality approaches as it does not cause systemic immunosuppression, and radiotherapy may prime the immune system for antigen release and help in reversing T-cell exhaustion. The immunomodulatory effects of radiotherapy are well established and include normalization of tumor vasculature, improved T-cell homing [9], destruction of immunosuppressive stromal cells in the tumor microenvironment, enhanced neoantigen generation and cross-presentation, amongst many others.

Furthermore, radiotherapy in combination with immunotherapy can induce response to therapy in patients who previously failed to completely respond to checkpoint blockade alone [10] – such as in the case discussed. This suggests that radiotherapy may stimulate a response to immunotherapy and re-activate the immune system. When multimodal immunoradiotherapy was reproduced in murine models, resistance was related to upregulated tumor PD-L1 expression and T-cell exhaustion. Therefore, these pre-clinical results suggest that a combination of immune checkpoint inhibition and radiotherapy may allow for increased immune target diversity, and thereby improve tumor response. Taken together, these results provide a logical clinical rationale for combined immunoradiotherapy approaches.

Sequencing of radiotherapy and immune checkpoint inhibition

There is limited data surrounding the optimal radiotherapy dose and fractionation schedule when combined with immunotherapy to elicit systemic immune responses. Pre-clinical murine data suggest that combined radiotherapy and checkpoint inhibition can improve survival [11]. Existing data is conflicting regarding standard dose and fractionation (<2 Gy) vs higher dose in terms of immune priming and tumor/abscopal responses. Some reports show that the combination of anti-CTLA-4 antibody therapy and three to five fractions of radiotherapy delivered at 6-8 Gy, but not at 20 Gy in a single fraction, enhanced abscopal responses and were associated with CD8+ T-cells density [12]. However, when recapitulated in a murine melanoma tumor model, ablative radiotherapy (such as 20 Gy, above) was capable of generating CD8+ T-cell-mediated responses [13]. Clearly, more data is necessary and further studies regarding the timing, dose, and fractionation of radiotherapy and immunotherapy are required.

## Conclusions

Herein we discussed the case of a patient with widespread metastatic melanoma with durable, long-term disease control. This report, amongst others, demonstrates that there may be a subset of patients with metastatic melanoma for whom systemic disease control is possible and for whom aggressive local therapy can induce durable, systemic immune responses which significantly impacts clinical survival outcomes. There is growing data suggesting that combined immunoradiotherapy approaches with checkpoint blockade combined with ablative radiotherapy may optimally prime the immune system through a variety of mechanisms; however, ongoing studies are necessary. Considering the disease response in this patient highlights the need for improved prognostic and stratification techniques to identify patients most likely to benefit from aggressive combined modality therapy for maximal clinical impact.
